# The Role of Red Blood Cells in Enhancing or Preventing HIV Infection and Other Diseases

**DOI:** 10.1155/2013/758682

**Published:** 2013-10-10

**Authors:** Modisa S. Motswaledi, Ishmael Kasvosve, Oluwafemi O. Oguntibeju

**Affiliations:** ^1^University of Botswana, Department of Medical Laboratory Sciences, Faculty of Health Sciences, Gaborone, Botswana; ^2^Oxidative Stress Research Centre, Department of Biomedical Sciences, Faculty of Health & Wellness Sciences, Cape Peninsula University of Technology, Bellville 7535, South Africa

## Abstract

*Aim*. To highlight the apparently neglected role of erythrocyte antigens in the epidemiology of infectious diseases, especially HIV, with the prime objective of stimulating research in this area. *Method*. A literature search was performed on the PubMed for relevant papers from 1984 to 2013, the era covering active HIV research. This was achieved by using the phrases “erythrocyte blood groups HIV” (81 papers) or “red cell antigen, blood groups, and HIV” (60 papers). A manual Google Scholar search was done and supplemented by original papers referenced by various authors. However, the review was limited by the relative scarcity of papers on the subject, and only papers written in English were reviewed during the period October 2012 to September 2013. *Results*. Many communicable and noncommunicable diseases are associated with specific blood groups. Examples of these diseases are discussed in detail. HIV has been shown to bind to erythrocytes, and candidate erythrocyte-binding molecules and mechanisms are also discussed. Moreover, erythrocyte-HIV binding is associated with increased viral infectivity, thus, underscoring the need to study this phenomenon and its implications for HIV epidemiology. *Conclusion*. Erythrocyte antigens may be important in the pathogenesis and epidemiology of many diseases, including HIV.

## 1. Introduction 

Blood groups have been known to exist for centuries ever since man entertained the possibility of replacing blood loss through transfusion. They are responsible for determining compatibility of blood in transfusion medicine and are also responsible for fetal loss in hemolytic disease affecting the fetus or newborn [1]. 

Blood groups are inherited as Mendelian codominant [2] traits and should be expected to occur in somewhat comparable frequencies in the human race. However, differences do exist in the distribution of blood groups in various human populations. This has been largely attributed to selection pressure as endemic diseases appear to have a predilection for selected blood groups leading to the demise of individuals bearing those susceptible blood group antigens [3, 4]. But certainly, there must be more to blood groups than just causing problems in blood transfusion and predisposing to myriad self-annihilation prospects. 

On the other hand, HIV has emerged as one of the major public health concerns of the 21st century. Some genetic factors have been cited as contributors to HIV susceptibility or resistance, among them blood groups such as ABO/Rh [2, 5–8], Duffy [9–12], and P^k^ [13]. However, the matter has not been without controversy as some investigators have reported findings to the contrary [14, 15] against what appears to be conclusive evidence of red cell interaction with the virus [9, 16–18]. Blood groups, therefore, appear to have a contribution to public health, at least in the area of infectious disease, which makes it imperative to synthesize available knowledge in an attempt to decipher the extent to which RBC antigens are involved in HIV epidemiology and to unveil avenues for future research; here follows a synthesis of current research on the beneficial and detrimental effects of red blood cell (RBC) antigens with probable molecular explanations.

## 2. Physiologic Functions of RBC Antigens

The physiologic functions of most blood group antigens are unknown [19]. Few antigens, however, such as the Kidd (urea transporter), Diego (anion exchanger), Colton (water channel), Cromer (Decay Accelerating Factor), and Duffy (chemokine receptor) have had their physiologic functions somewhat elucidated [20, 21]. The importance of such functions remains a matter of debate though, since individuals who are negative for these blood group antigens live an apparently normal life [22]. 

A lot has been documented about the Duffy blood group as a chemokine receptor, a function that has led to its designation as the Duffy antigen receptor for chemokines (DARC), and to the investigation of its potential role in modulating the inflammatory response [9, 10, 23–25]. Chemokines are important for recruiting white blood cells to the site of infection or inflammation by binding to their receptors. However, HIV also uses these chemokine receptors as coreceptors for its entry into permissive cells. It is known that R5 viruses utilize CCR5, while R4 viruses use CXCR4 as a co-receptor. The binding of HIV to the coreceptor and its subsequent entry into the cell are inhibited by chemokines which act as competitors for the binding site [9, 10]. The Duffy antigen receptor for chemokines binds a wide range of chemokines and thus competes with white blood cells for chemoattractants as well as increase cell exposure to HIV infection. DARC also binds other inflammatory mediators such as interleukin 8 (IL-8), RANTES and monocyte chemoattractant protein-1 (MCP-1) [26–28], all of which are important in contributing to a successful defense against pathogens.

Red blood cells also bear the CD44 antigen, a ligand for hyaluronic acid. Binding of hyaluronic acid to this RBC antigen would have the significance of dampening its inflammatory effect. As in the case of the Duffy antigen binding chemokines, this erythrocytic property may be important in absorbing excess stimulant signals intended for inflammatory cells, thereby modulating immune responses [21]. In cases where the stimulant is produced in small amounts, it is plausible that erythrocyte antigens, by their sheer numbers, may out-compete the intended cells, leading to an immunological hyporesponse.

Human red blood cells not only transport oxygen and carbon dioxide, but are also important in the elimination of many molecules from circulation, including products of immunologic reactions. RBC possess adhesion molecules that bind complement fragments [29] and are therefore important for capturing and delivering such complexes to the reticuloendothelial system for elimination from circulation. In this regard, Horakova et al. [29] investigated the binding of HIV immune complexes to erythrocytes. Their investigations led to the conclusion that at least three mechanisms are involved. These being, the binding of complement-opsonized immune complexes via CR1, direct binding of the virus in a complement-dependent manner but in which specific antibodies were not required, and a third mode in which complement was not required at all. Their conclusion was that the third mode of binding might be instrumental in the spread of infection at the time of primary infection when specific antibodies would not have been formed yet.

Some RBC antigens are also known to bind inflammatory mediators with potential to affect innate immune responses [21, 23]. These RBC antigens absorb and limit the amount of chemokine stimulant activity [27]. Indeed it has been demonstrated that mice lacking the Duffy antigens exhibit a greater inflammatory response to CXC and CC chemokines compared to homozygotes or heterozygotes [30]. This is consistent with the role of Duffy as a “chemokine sink” created by chemokine-binding sites on DARC(+) erythrocytes. But, paradoxically, the absence of the Duffy antigens has also been associated with a low neutrophil count [11, 27]. Given the observation that DARC also binds HIV and is capable of transfecting CD4 cells [10], it is probable that HIV-binding to erythrocytes represents an action of various molecules acting in synergy. 

## 3. Blood Group Disease Associations

On the pathological front, however, certain blood groups have been associated with diseases. Individuals of blood group A, for example, are known to be more susceptible to coronary heart disease (CHD) independent of known risk factors than other ABO blood groups [31]. This is further corroborated by the observation that group A individuals also have higher levels of low density lipoprotein (LDL) cholesterol [19]. Although the molecular aspects of this observation still remain to be elucidated, the observation implies that, this antigen has potential to either influence synthesis or inhibit the natural metabolism of these lipids, thus, predisposing individuals to CHD. The same study, on the other hand, reported a lower risk for this condition among group O individuals.

Additionally, blood group A and B are known to be highly susceptible to thrombotic disorders in contrast to group O individuals who are more at risk for bleeding than thrombotic events [32–34]. This observation has been attributed to higher levels of Factor VIII and von Willebrand factor in A and B individuals, while the levels may be as low as 30% in group O individuals. Blood groups A and B antigens have been demonstrated on these coagulation molecules and are thought to prolong their half-life, leading to higher concentration in non-O individuals [35].

Blood group A has also been associated with malignancies such as cancer of the ovary, cervix, rectum, breast, and stomach and leukemia [19]. This is thought to be due to an abundance of high-affinity binding sites for epidermal growth factor (EGF) on blood group A red blood cells compared to blood groups O and B [36]. Since blood groups may be expressed on tissues other than red cells [21], it is plausible that the binding of EGF to these binding sites may indeed promote cancer development. Conversely, some malignancies have been observed to positively or negatively modulate blood group antigen expression to varying degrees depending on the anatomical site [19]. Thus, gastric carcinomas were found to be associated with increased expression of ABH and Le^a^ antigens while colonic cancers were associated with expression of Le^b^ and low expression of the other antigens. In other studies, enhanced fucosyltransferase activity was reported in endometrial cancer leading to unexpected expression of Le^a^, Le^b^, and H antigens [37, 38]. 

The Duffy blood group is probably one of the most studied blood groups in relation to disease association. Individuals who are homozygotes or heterozygotes for the Duffy antigens, Fy^a^ or Fy^b^, are vulnerable to malaria parasites *Plasmodium vivax* and *Plasmodium knowlesi*. Individuals that lack both Duffy antigens, Fy(a-b-), and therefore lack the receptor for malaria parasites are constitutively resistant to these forms of malaria [21, 39].

## 4. Blood Groups in HIV and Other Viral Infections

### 4.1. Blood Groups in HIV Infection

Most research on HIV has focused on cells of the immune system to the exclusion of viral interactions with red blood cells. However, emerging evidence suggests that red blood cells may be important in the pathogenesis of HIV as they enhance viral infectivity by binding free viruses [16] as well as viral immune complexes [16, 29, 40], and through such binding transfect HIV-susceptible cells [29, 40].

HIV infection has been reported to occur in select blood groups in some regions of the world. A study by Sayal et al. in India reported a preponderance for infection in group O Rh(D)-positive men and least among groups B positive and D-negative ones. However, a close examination of the results reveals insufficient statistical analysis rendering the differences statistically insignificant. Similar studies by Nneli et al. [41] and Dirisu et al. [8] suffered similar deficiencies. In these studies, group O positive individuals were thought to be highly susceptible, but again the studies lacked the statistical rigor to indicate the level of significance and have been contradicted by other investigators [14]. A statistical interrogation of the data in each of the above case does not support the conclusion of statistical significance.

It would then appear that current scientific information does not support a potential role for ABO blood groups in HIV infection. In fact, evidence from other studies would suggest the contrary for group O individuals. Since HIV virions have been shown to acquire the blood group antigens of the infected individuals [2, 6, 42], such virions would be neutralized by naturally-occurring antibodies in group O individuals, thus offering protection in blood group-discordant couples [2]. It is noted, however, that this protection will not be available if the source of infection was of a similar blood group. Moreover, given the apparently uniform risk of infection among ABO blood groups, it is doubtful if this neutralization is of any clinical consequence, especially with reference to HIV-1 infection. It remains to be demonstrated then whether HIV from blood group A or B is able to infect group O CD4 cells.

In a separate study, Abdulazeez and colleagues [7] working with HIV-1 and -2 reported a higher prevalence of HIV-2 (71.4%) compared to HIV-1 (7.1%) in the AB blood group and that Rh(D) positive (97.8%) was more susceptible than D negative (2.2%). However, these findings are yet to be confirmed by other investigators.

The lack of direct empirical evidence for ABO blood groups does not, obliterate the possibility of associations in other blood groups. Since secreted blood group substances can be adsorbed onto lymphocyte membranes [2, 6, 42], the presence of these antigens could potentially alter cell behavior. Blood group antigens, being glycoproteins and glycolipids, are highly charged molecules that are bound to affect their molecular microenvironment, including protein conformation and receptor/CD4 localization and function [13, 19]. Glycosphingolipids and glycoproteins have in fact been demonstrated to facilitate fusion of HIV-1 with CD4 cells by independent investigators, thus, acting as alternative co-receptors for the virus [17, 18]. There exists a theoretical possibility, therefore, that membrane-bound blood group substances on CD4-positive cells may thus affect the affinity of viral-binding proteins and viral infectivity, promoting or diminishing cell susceptibility to infection. Apparently, both the promotive and inhibitory effects of blood groups in HIV infection have been documented. The Duffy antigen receptor for chemokines (DARC) has been reported as a binding site for HIV-1 on red blood cells, which binding increases infectivity for permissive cells [10, 12]. Although some investigators have refuted the role of this antigen in HIV-1 infection [15, 43], the preponderance of evidence is heavily in favor of this role, including recent findings of sequence similarities between HIV-1 V3 loop and the *Plasmodium vivax* Duffy-binding protein, both of which bind to DARC. 

Furthermore, the Duffy Null phenotype has been associated with increased susceptibility to HIV infection among African Americans, elevating the odds of acquiring HIV by as much as 40%, while those negative for the antigen were observed to exhibit slower progression to AIDS [44]. Although these findings were later refuted by other investigators [43, 45], there is persuasive evidence suggesting the role of DARC in binding the virus and facilitating transfection of permissive cells [9–11]. These reports were given further credibility by findings that the Duffy-binding protein of *Plasmodium vivax* bore sequence similarity with the V3 loop of HIV1 [9].

He et al. [12] reported that the DARC+ phenotype was associated not only with higher rates of HIV-1 infection, but also that DARC+ red cells transfected HIV-susceptible cells with X4 variants more efficiently than it did with X5 variants. Blocking of the DARC with monoclonal antibodies or other natural ligands significantly reduced viral binding, thus, confirming the role of DARC in the interaction. However, the blocking of the DARC did not totally obliterate HIV binding to erythrocytes, thus, suggesting possibilities of involvement of other binding molecules as hypothesized by Beck et al. [16]. Similar findings were also reported from a study involving commercial sex workers in South Africa, where investigators found that a Duffy-null phenotype with low neutrophil counts was predictive of HIV-1 seroconversion [11].

Another such molecule is the P^k^ RBC antigen, which has been reported to confer resistance to HIV infection in lymphocytes *in vivo *and* in vitro*. In this Canadian study, the level of expression of P^k^ antigen on peripheral mononuclear cells (PBMC) was positively correlated with antiviral protection [13]. The P^k^ antigen has a low frequency (one in a million) [13] among Caucasians and is thought to have contributed to reduction of HIV spread among such populations. The CCR5Δ32/Δ32 is another mutation that is prevalent in the same population at 1–3% [12] and is known to confer resistance to HIV infection. While it has been argued that the lack of P^k^ among Africans may not account for the difference in the epidemiology of HIV in Sub-Saharan Africa (given its low frequency among Caucasians), one could consider the combined effect of both genes in the same population and the number of contacts that could have been generated if such genes were not operational, compared to a population that lacks both.

In addition, Beck et al. demonstrated that human red blood cells bind HIV virions independent of antibody or complement. The RBC selectively bound infectious HIV virions as opposed to other viral components and removed all infectivity from the suspension medium. The HIV-binding to erythrocytes was calcium-dependent but complement and CD4-independent. Moreover, RBC-bound virions were 100-fold more infectious to CD4 cells than cell-free particles [16], leading them to conclude that red blood cells could constitute a viral reservoir for transinfection of CD4 cells. The Beck report [16] essentially confirmed earlier findings by Hess et al. [40] of a pool of HIV viral RNA associated with RBC in HIV-1-infected patients. These included patients in whom plasma viraemia had been suppressed to undetectable levels for several months with antiretroviral drugs. Their investigations revealed higher numbers of RNA copies per mL of whole blood in the RBC than white cell preparations of the same patients, suggesting that the bulk of the cell-bound virus circulates on RBC rather than white blood cells (WBC). RBC-bound HIV was also able to infect HeLaT4 cells. The number of erythrocyte-bound HIV copies significantly correlated with disease severity [40]. The total sum of these findings is that RBCs are important elements for consideration in HIV infection and could potentially influence epidemiology and clinical progression.

In order to deal with this reservoir of infection, there is a need to understand the nature of the RBC-HIV binding, especially now that there is evidence linking certain blood groups to susceptibility or resistance to HIV [2, 13, 17, 41]. 

### 4.2. Blood Groups in Other Viral Infections

The binding of viruses by red blood cells, including specific binding to blood group antigens is not unique to HIV, although the mechanisms involved may differ. Noroviruses have been observed to selectively bind to group A, H, and difucosylated Lewis blood groups [46, 47], and only volunteers who were secretors of these antigens were found to be susceptible to norovirus gastroenteritis [4]. Although this binding was not directly related to red blood cells, it nevertheless demonstrated viral affinity for a red blood cell antigen expressed on gastric mucosal cells and led to the conclusion that the groups A, H, and Le^b^ antigens act as viral receptors for the virus [4, 48]. 

## 5. Anemia

Anaemia is the most important prognostic indicator in AIDS patients [49–51]. A large-scale study involving over 4000 patients established an increasing hazard ratio of 1.42, 2.56, and 5.26 for mild, moderate, and severe anaemia, respectively [50]. Various mechanisms have been suggested, including bone marrow suppression by various cytokines, toxic depletion by the virus, and immune destruction following sensitization with viral proteins [52–54]. However, the degree of anaemia has not been correlated with the amount of erythrocyte-bound HIV (E-HIV), a phenomenon that might account for the autoimmune haemolytic anaemia that commonly occurs in HIV-infected individuals. Of interest is the observation by Martins-Silva et al. that HIV infection alters red cell and lymphocyte membrane fluidity and membrane protein activity and brings about changes in transmembrane calcium transportation [55]. These changes may disrupt erythrocyte membrane stability, thus, promoting haemolysis and ultimately anaemia. 

Blood group antigens have also been reported to occur on trans-membrane transport channels [21]. These transport channels have an influence on membrane structure and integrity and HIV binding to these transport channels may explain some of the mechanisms that contribute to membrane instability with consequent haemolysis and cytopaenias in HIV patients. Binding of R5 viruses to CCR5, for example, has been shown to elicit phospholipase C production [56]. Furthermore, others have reported that phospholipase C is translocated to the periphery of activated natural killer (NK) cells with possible role in cell-mediated cytotoxicity [57]. However, the role of this enzyme in haemolytic processes in HIV patients remains a matter for further enquiry.

The haemolytic effects of phospholipase C could be further aggravated by the presence of lipolytic enzymes emanating from the various opportunistic infections. Many bacteria produce this lytic enzyme including *Mycobacterium tuberculosis*, a common opportunistic pathogen in HIV patients [58–61].

## 6. Conclusion

The role of ABO blood groups in infectious and non infectious diseases is well established. Much scientific evidence also exists to support the role of erythrocytes in binding viruses and facilitating infection of permissive cells. Some blood groups such as the Duffy type are implicated as candidate molecules. Other molecules such as the P^k^ blood group which are present in Caucasian populations have been shown to confer resistance to HIV infection. The study of blood groups in HIV infection may shed light on the dynamics of HIV epidemiology, especially in hard-hit SubSaharan Africa.

Finally, HIV continues to take a toll on the health and economies of the world especially the developing countries. To date a cure for this virus continues to be elusive. However, a virtually complete remission has been attained in one patient following a bone marrow transplant of a CCR5Δ32/Δ32 mutant cell line [62]. However, despite this milestone, donors with this mutation are still far too rare and limited to European populations. Very few studies have focused on the role of blood groups in preventing or enhancing HIV infection, and these few had the limited scope of only looking at the ABH and Rh(D) blood groups. Africa suffers from a lot of malignancies that require total body irradiation and bone marrow reconstitution, some of them linked to HIV infection. The occurrence of HIV-resistant blood groups may avail bone marrows that may as well be an answer to HIV eradication in such cancer patients.
